# The experiences of speech-language therapists providing telerehabilitation services to children with autism spectrum disorder

**DOI:** 10.4102/sajcd.v69i2.917

**Published:** 2022-08-31

**Authors:** Saira B. Karrim, Penelope S. Flack, Urisha Naidoo, Stephanie Beagle, Abigail Pontin

**Affiliations:** 1 Discipline of Speech-Language Pathology, School of Health Sciences, University of KwaZulu-Natal, Durban, South Africa

**Keywords:** autism spectrum disorder, experiences, telerehabilitation, speech language therapist, South Africa

## Abstract

**Background:**

There has been an increased emergence of the use of telerehabilitation by speech-language therapists (SLTs) in South Africa since the COVID-19 pandemic in 2020.

**Objectives:**

To explore the criteria that SLTs use when recommending telerehabilitation for children with autism spectrum disorder (ASD), the technical skills required, strategies used by SLTs, the restrictions encountered when conducting telerehabilitation and the views of SLTs on telerehabilitation in comparison to face-to-face therapy for children with ASD.

**Method:**

A descriptive, phenomenological, qualitative study design was utilised. Purposive and snowball sampling techniques were employed. Six SLTs from the private sector, who had experience providing telerehabilitation to children with ASD, were recruited from three provinces in South Africa. Data were gathered via semistructured online interviews and analysed using thematic analysis.

**Results:**

Two out of five themes that emerged from this study are presented in this paper, i.e. approaches to telerehabilitation and the benefits of telerehabilitation. Results revealed that telerehabilitation was used to provide assessment and therapy during the COVID-19 pandemic lockdowns as an alternative method of service delivery. Assessment and treatment strategies included synchronous and asynchronous methods, family collaboration, social stories, frequent breaks and interactive sessions. Telerehabilitation reduced the client’s and SLT’s travel costs and increased caregiver and clinician satisfaction. Client progress and increased awareness of SLT were viewed as further benefits.

**Conclusion:**

Telerehabilitation was found to be beneficial to most children with ASD, and in most cases, the benefits far outweighed the challenges encountered. Clinical implications included the need for caregiver support in facilitating effective carryover, an increase in SLTs’ knowledge and the opportunity to provide services to a broader geographical range. Limitations of the study are included.

## Introduction

Speech-language therapists (SLTs) are core members of the interprofessional team responsible for the management of children with autism spectrum disorder (ASD), a neurodevelopmental disorder characterised by restrictive and repetitive behaviour, impaired social communication skills and sensory difficulties (Lord et al., 2020). Approximately 270 000 people in South Africa are diagnosed with ASD (Van Biljon, Kritzinger, & Geertsema, 2015), with a projected 5000 new cases per year (Springer et al., 2013). Children with ASD may be able to access support services through specialised educational placements in either the public or private sectors. However, these are limited, resulting in significant numbers of children not having access to necessary support (Bateman, 2013). In South Africa, historical, social, political and economic factors hamper equitable access to care (Malcolm-Smith, Hoogenhout, Ing, Thomas, & De Vries, 2013). This is compounded by the extreme shortage of healthcare workers to serve a rapidly growing population, particularly in the field of speech-language pathology, where a 1:18 000 SLT-to-patient ratio is likely (Health Professions Council of South Africa, 2018).

One answer to expanding access to services is telerehabilitation (Mars, 2011). This refers to the use of electronic information and telecommunication technologies to provide clinical services to patients who have difficulties in accessing health services or attending face-to-face therapy (Bettger & Resnik, 2020; Wacker et al., 2013). Telehealth, which is an umbrella term for modalities that include telerehabilitation (Bettger & Resnik, 2020), is becoming an increasingly viable alternative model of service delivery, especially to ensure access to healthcare in situations where traditional face-to-face visits are difficult. Telecommunications technology is used to provide services, which can happen in real time (synchronously) or through prerecorded videos or voice notes (asynchronously) or through a hybrid approach (American Speech and Hearing Association, 2019). This technology may include social media platforms and online meeting applications, typically requiring both audio and video technology (Aggarwal, Patel, & Ravi, 2020). Synchronous telerehabilitation is the most common method used by SLTs. The clinician and the client communicate and interact directly using videoconferencing via the phone, computer or laptop (Khoza-Shangase, Moroe, & Neille, 2021; Simacek, Elmquist, Dimian, & Reichle, 2021; Solomon & Soares, 2020). This allows for SLTs to provide the caregiver and child with instant feedback, instructions and positive reinforcement as the intervention is provided to the child (Simacek et al., 2021). On the other hand, asynchronous telerehabilitation allows a caregiver to complete the therapy activity at a convenient time and send or e-mail images, recordings and monitoring charts to the SLT for analysis and interpretation (Hill & Breslin, 2016).

In the early months of 2020, the coronavirus disease 2019 (COVID-19) pandemic swept across the globe, and efforts to contain the spread of the virus included various forms of lockdown. In South Africa, the government declared a national lockdown at the end of March 2020. Although people were allowed to shop for necessities and seek emergency medical care, all other forms of face-to-face contact were discouraged. Even when the lockdowns eased, many people felt unsafe returning to face-to-face contact in schools and healthcare centres. Alternative forms of service delivery then became paramount, and telerehabilitation was one solution. However, little was known about how SLTs could use telerehabilitation to provide health services to children who present with ASD.

## Literature review

Speech-language therapists play a critical role in the multidisciplinary team, both in terms of contributing to the differential diagnosis and in providing support to children with ASD (Dillenburger et al., 2014). Early identification, followed by effective intervention, can reduce the burden of ASD upon the child, the family and the community. The SLT’s particular role is to support the development of skills that will help the children communicate their wants and needs in the most functional way, as well as exchange ideas and comprehend others’ intentions in various settings (Parsons, Cordier, Munro, Joosten, & Speyer, 2017). The role of SLTs extends to supporting both verbal and nonverbal communication, providing augmentative and/or alternative forms of communication and training others involved in the care and education of children with ASD. Ongoing, consistent intervention is therefore crucial. The disruption to face-to-face interactions caused by the COVID-19 pandemic resulted in SLTs rapidly exploring alternative forms of service delivery, such as telerehabilitation, to ensure continued access to service as far as possible (Campbell & Goldstein, 2021; Elsami Jahromi, Farokhzadian, & Ahmadian, 2021; Kraljević, Matić, & Dokoza, 2020; Sutherland et al., 2021; Theodoros, 2008; Tohidast, Mansuri, Bagheri, & Azimi, 2020).

Although telerehabilitation (referred to as telehealth and telemedicine in some literature) has been gaining traction globally for the past few decades (Law, Dornstauder, Charlton, & Gréaux, 2021), it is a relatively new and still emergent method of service delivery for the SLT in South Africa (South African Speech-Language-Hearing Association, 2020). Khoza-Shangase et al. (2021) reported limited literature on telerehabilitation in Africa. The Health Professions Council of South Africa (HPCSA) had provided guidelines for telerehabilitation as early as 2014 (Health Professions Council of South Africa, 2014). However, Hlayisi (2016) found that although SLTs appeared to understand telerehabilitation, few were using this approach. Prior to the COVID-19 pandemic, service delivery in SLT was typically one-on-one, in-person direct training and/or clinical service provision. Reluctance to embrace telerehabilitation may have stemmed from both perceived and real barriers, such as poor telecommunications infrastructure in South Africa, i.e. Internet or network availability, access to appropriate devices for both clinician and client or caregiver, the health professionals’ attitude towards telerehabilitation, ethical and legal issues and the challenges of multilingualism and multiculturalism (Chifamba, 2018; Hlayisi, 2016; Sinsky, Jerzak, & Hopkins, 2021). Nevertheless, there are significant benefits of telerehabilitation in SLT beyond the current focus on reduction of risk of infection through face-to-face contact, such as better access for people in rural and remote areas (Grillo, 2017), more cost-effective service delivery (Macoir et al., 2021) and more efficient use of time and resources (Elsami Jahromi et al., 2021; Khoza-Shangase et al., 2021); all are important considerations for the profession in South Africa.

Speech-language therapists face a range of difficulties when providing services to children with ASD through telerehabilitation, from accessibility to resources that are required for telerehabilitation, adequate training of SLTs and caregivers, active involvement of the caregiver and the child and the appropriateness of the environment where the therapy is conducted (Edwards, Stredler-Brown, & Houston, 2012).

Firstly, telerehabilitation requires the SLT and caregiver to have compatible devices to facilitate communication during therapy. Such devices may include laptops, computers, cell phones and telephones (Edwards et al., 2012), any of which can be used to support telehealth. Yet 5% of the South African population do not own or are not able to borrow a phone because of challenges such as theft, cost of the device and lack of sufficient literacy for the use of smartphones; high data costs and access to electricity also impact on access to telehealth services (McCrocklin, 2021). Use of phones rather than laptops or iPads is not favoured by clinicians because of the small screens, which potentially compromise the quality of the image of the clinician and/or resources being presented (Gibbs, Cai, Aldridge, & Wong, 2021).

Secondly, the necessity for the caregiver to be able to troubleshoot the device, programme or connectivity problems should they arise requires some training and upskilling, particularly in South Africa where it is not uncommon in rural and remote areas for the primary caregivers to be elderly grandparents, who are more likely to be illiterate and/or have poor technological literacy (Gibbs, 2021; Khuluvhe, 2021; Le Roux, 2020; Rao, 2018).

Thirdly, the caregiver should ideally be actively involved in the sessions. This poses a challenge in terms of both appropriate training regarding the technology and the appropriate behaviour (for example, feedback and monitoring) during the session. Parent training is particularly important when working with children with ASD (Burgoyne & Cohn, 2020). Parent training is one way for the SLT to reduce cultural barriers associated with telerehabilitation in children with ASD and their families (Alkhalifah & Aldhalaan, 2018). However, a challenge linked to this is the caregiver’s availability, which may limit times for synchronous sessions.

Reports from SLTs using telerehabilitation indicate that both quality and quantity of responses from the child are compromised (Simacek, Dimian, & McComas, 2017). In synchronous sessions using Zoom technology, for example, time lags because of poor connectivity or imbalances in the way multiple voices are amplified can result in a breakdown in communication or compromise the feedback. This feedback is necessary to assess the child’s receptiveness to the treatment plan and the strength of the cooperation (Burgoyne & Cohn, 2020). Nonverbal behaviours and facial expressions are also more limited as these are impacted both by the technology and the equipment the child and the SLT are using. Camera positioning is critical to ensure the child is able to move around the chosen space without compromising the SLT’s view or sound quality (Burgoyne & Cohn, 2020). Young children with complex communication needs especially may have negative responses to equipment such as headphones (Grogan Johnson et al., 2013). Johnsson and Bulkeley (2021) suggest that children with ASD usually present with behavioural difficulties which interfere with their ability to engage with telerehabilitation effectively.

In contrast to the challenges and negative experiences reported earlier, Gibbs et al. (2021) found that clinicians working with adults with ASD and their families shared positive technology experiences. Nevertheless, they too expressed concerns about how the technology negatively impacted their assessment experience. In a telerehabilitation assessment session, significant features of client behaviour may not be as noticeable to the clinician, with eye contact highlighted as the most challenging behaviour to observe whilst the easiest skills to assess were verbal and conversational skills (Gibbs et al., 2021).

### Strategies used in telerehabilitation

Recommendations for successful telerehabilitation are highlighted in the literature. Having a flexible plan, a selection of toys and props, limiting session times and breaking sessions into therapeutic games have proven to be effective when working with children with ASD and their families (Burgoyne & Cohn, 2020). Shorter sessions also allow for additional time to work with the caregiver, whereas with older children the clinician can adapt games to the digital platform or make use of various applications where relevant (Camden et al., 2020). Therefore, caregivers need to be involved in the selection of the resources. The use of telerehabilitation allows for collaboration between the SLT and the child’s family to develop appropriate therapy goals to be targeted, as well as activities tailored to the individual child. The use of auditory, verbal and visual strategies are considered the most effective strategies when providing services to children with ASD (Ngcobo, 2015), as this combination provides the SLT, the caregiver and child with the most effective learning environment.

Camden et al. (2020) reported that telerehabilitation is usually found to be more successful with children older than 6 years of age. Late diagnosis is particularly prevalent in South Africa, and in rural communities the average age for a child diagnosed with ASD is 7 years (Solomon & Soares, 2020). In South Africa, early intervention services for children with ASD are limited in the public sector because of the limited number of professionals and collaboration between professionals and families (Guler, De Vries, Seris, Shabalala, & Franz, 2018). Popich, Louw and Eloff (2006) found that 84% of parents of children with ASD were aware of their children’s delayed communication abilities but reported that they did not have access to appropriate resources to target these delays. Although this study was conducted more than 15 years ago, little has changed, and healthcare services currently provided to children using the face-to-face model are insufficient for the increasing number of children with ASD and their families. Thus, despite the current focus on telerehabilitation resulting from the COVID-19 pandemic, even after all restrictions are lifted, there is likely to be a significant need for alternative forms of service delivery such as telerehabilitation for children with ASD.

### Rationale for the study

There is insufficient information available in the literature regarding the use of telerehabilitation by SLTs when working with children with ASD and even less focused on children in sub-Saharan Africa (Goldstein, Klaiman, & Willliams, 2017; Guler et al., 2018; Khoza-Shangase, 2021; Sutherland et al., 2018). Most of the studies conducted globally regarding the use of telerehabilitation and children with ASD have not provided detailed information regarding the strategies and techniques used by SLTs when conducting assessment and intervention. These include strategies used to build rapport with the child and caregiver, assessment, providing comfort, showing empathy and monitoring skills that are impacted by physical and cognitive difficulties when co-occurring with ASD (Goldstein et al., 2017). This gap in the literature extends to resources and tools. Tools used during ASD evaluation have not been translated into telerehabilitation format, and some of the strategies used during telerehabilitation are not evidence-based. Furthermore, there are currently inefficient policies guiding the use of telerehabilitation to manage behavioural difficulties in children with ASD (Johnsson & Bulkeley, 2021; Solomon & Soares, 2020). A lack of evidence-based practice can result in SLTs not feeling confident enough to provide telerehabilitation services to children with ASD, impacting their experience utilising it. Over the past decade, telerehabilitation studies in SLT have focused mainly on providing instructions to parents, caregivers and teachers. Therefore, the focus of this study is on the experiences of SLTs providing telerehabilitation services to children with ASD.

## Methodology

### Aim

To explore the experiences of SLTs providing telerehabilitation services to children with autism spectrum disorder.

### Objectives

1. To explore factors that impact on the decision to recommend telerehabilitation for children with autism spectrum disorder.
2. To describe the SLT’s approach when conducting telerehabilitation for children with autism spectrum disorder.
3. To describe the views of SLTs regarding the challenges and benefits of telerehabilitation for children with autism spectrum disorder.

### Research design

A qualitative descriptive phenomenological approach was used. According to Pathak, Jena and Kalra (2013), researchers use the qualitative approach when they are gathering in-depth data or information about people’s daily life experiences. According to Sutton and Austin (2015), qualitative research allows the researchers to gain more insights and precise knowledge about the phenomenon that was the participants’ daily experiences using telerehabilitation with children with ASD and their professional role of assessing and supporting children with ASD.

### Study setting

Speech-language therapists from anywhere in South Africa could participate in this study. The online nature of the study did not restrict participation to a specific province. Because of the COVID-19 pandemic, this study was conducted online and ensured no direct contact between the researchers and the participants, maintaining safety and social distancing (Dodds & Hess, 2020).

### Participants

Purposive, snowball sampling was used. Initially requests for participants were sent to both the Department of Health and the Department of Education as well as online social media groups. As gatekeeper permission was not obtained timeously from the Departments of Health and Education in time to complete data collection, only those participants who were recruited via online and personal social media groups were included. The groups were closed Facebook groups for SLTs or speech-language audiologists and WhatsApp groups of SLTs that served for sharing ideas, information and resources. Six participants met the inclusion criteria, which were registration with the HPCSA as SLTs or speech-language therapists and audiologists, a minimum of 6 months of experience with telerehabilitation and experience using telerehabilitation as well as face-to-face therapy with children presenting with ASD aged 2–12 years in South Africa. All participants came from the private sector and had between 4 and 15 years of experience as SLTs; all but one started using telerehabilitation from February 2020. Table 1 illustrates the description of the participants.

**TABLE 1:** Description of the participants.

Code	Province	Years of SLT experience	Practice context	Started telerehabilitation
P1	Western Cape	4 years	Private practice – education	April 2020
P2	Gauteng	6 years	Private practice – education and health	Mid-April 2020
P3	KwaZulu-Natal	15 years	Private practice – education and health	March 2020
P4	Gauteng	13 years	Private practice – education	February 2020
P5	KwaZulu-Natal	9 years	Private practice – education	March 2020
P6	Gauteng	10 years	Private practice – education	2012

SLT, speech-language therapist.

### Procedures

Semistructured interviews were conducted with each participant online via Zoom. This was an appropriate alternative to face-to-face interviews because of the restrictions on personal research interviews imposed by the COVID-19 pandemic. Owing to limited participants, the pilot study was done with a qualified SLT who conducted telerehabilitation as a final year student at the University of KwaZulu-Natal and had 9 months of experience as a qualified professional at the time of the study. The purpose of the pilot study was to test the data collection tool and process, following which changes were made. Therefore, data and participant information were excluded from the main study.

The results of this pilot study revealed a need for frequent follow-up of participants to schedule interviews, and there were some minor adaptations to the interview schedule, such as rephrasing of some questions to facilitate better understanding of the questions. Potential participants identified by the researchers were provided with an information letter about the nature of the study, benefits of the study, their role and details regarding data collection and distribution. This information letter, which covered confidentiality, provided participants with detailed information to allow them to make informed decisions about whether to participate in the study or not. Participants’ rights to self-determination and withdrawal from the study were emphasised. An e-mail with screening questions was sent to interested participants to identify if they met the inclusion criteria. For example, ‘have you provided telerehabilitation services to children with ASD?’

Once the participants who met the inclusion criteria were identified, they were asked to complete the informed consent document. This was distributed and collected via e-mail. Each interview lasted approximately 30–45 min. The interviews were recorded on Zoom and on a Dictaphone, uploaded and saved in password-protected electronic files on the researchers’ laptops immediately after the interview. Participant identity was protected by assigning codes to all files. The semistructured interview schedule, containing five sections (general information, challenges of telerehabilitation, SLT attitudes, telerehabilitation strategies and telerehabilitation versus face-to-face therapy), was developed from relevant literature using the Kallio, Pietilä, Johnson and Kangasniemi (2016) framework. Data collection occurred from 02 November to 05 November 2021.

### Data analysis

The data were analysed according to the six phases of thematic analysis by Braun and Clarke (2012), which include familiarisation with the study, generating initial codes, searching for themes, reviewing of themes, naming of themes and reporting of results. The first phase required familiarisation with the transcribed data gathered from the interview. Otter.ai was used to assist with the written transcription of the data. However, it was important that the researchers personally transcribed each interview to begin the familiarisation process of the data. The interesting initial ideas were noted as the data were cleaned and reread. The second phase required generating codes on the data collected. These codes were smaller ideas or themes that were identified in the data. The data were then grouped into related ideas or themes that later provided ease of analysis for the next phases. Phase 3 required searching for themes by naming and interpreting the codes noted in the data. In this phase, the main overarching themes and subthemes within them were identified. Phase 4 of this analysis process focused on reviewing and refining the themes further. The themes were compared to the codes, and a thematic table was developed to highlight the main areas of the research question. Phase 5 focused on defining and naming the themes presented for the analysis. A supervisor review was conducted to confirm coding schemes and accurate analysis of the data, which resulted in the researchers editing the overarching themes of the research study.

### Trustworthiness

Credibility was ensured through the use of a pilot interview; member checking was conducted at the end of the interview with participants, whereby participants were allowed to alter, affirm or remove any statements they had made (Gibson & Brown, 2009). The findings of the member checks revealed that the researchers accurately captured their ideas and responses appropriately with no removal of any statements made. During data analysis, the transcripts were checked against video recordings for accuracy by the researchers. Dependability was ensured through the use of an audit trail to track decisions made at each stage of the research process. The researchers ensured confirmability by probing to gather richer data, participant screening and supervisor review of the transcripts to confirm coding schemes and accurate analysis of the data. Transferability was ensured with the use of purposive sampling to obtain a broad range of information that is contextually diverse, which was achieved by including participants who work in both health and education departments and who are from different geographical areas.

### Findings

**FIGURE 1:**
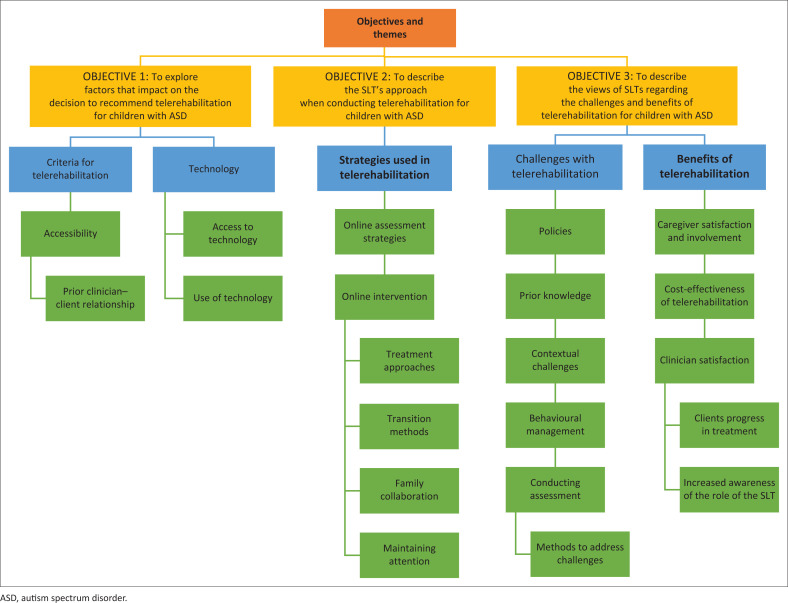
Overview of themes and subthemes that constitute the results of the study.

Multiple themes emerged under each of the objectives of the study. Figure 1 provides an overview of themes as they align to the objectives for the interest of the reader. However, only the themes linked to two of the objectives are discussed in this article, that is, objectives 2 and 3. The themes that are presented in this paper are strategies used in telerehabilitation and benefits of telerehabilitation. The themes linked to these two objectives were selected based on being the most relevant to SLTs providing or considering telerehabilitation.

### Strategies used in telerehabilitation

This theme discusses the online assessment strategies used, treatment approaches useful for telerehabilitation, transitioning methods, family collaboration and maintenance of the client’s attention.

#### Online assessment strategies

Three participants indicated the use of informal assessment strategies online, together with face-to-face assessments. Creative methods of getting as much information as possible included the use of video recordings. Participant 2 requested caregivers to send video recordings of the client in multiple contexts during everyday interactions. Thereafter, the Childhood Autism Rating Scale (CARS; Schopler et al. 1980, 1988) was used to analyse the videos. Participant 6 mentioned collaborating with caregivers and other healthcare professionals to gather assessment information about the client, including the use of classroom-based assessments to determine how the client was performing, and then based their goals on that:

‘A lot of the assessment is classroom based, how much of the classroom work do they know, what can they do? What can’t they do? And then from there it’s based on … our goals are based on functional elements. ’ (P6, SLT, Gauteng)

Unlike the participants earlier, Participant 4 conducted only synchronous telerehabilitation assessments. This participant reported using pictures presented on PowerPoint, scanning copies of formal tests and displaying them on the screen and play-based observation with real objects. Participant 4 further indicated that play was assessed in the same manner as face-to-face therapy, with the difference being that caregivers prepared the appropriate items and facilitated the assessment:

‘I get the parents to have matching toys and prepare spoons and plates. Then I say, “oh the lion is hungry and asleep” and the child automatically wants to feed the lion. So you assess play the same way that you would by getting the parents to prepare items for you.’ (P4, SLT, Gauteng)

#### Treatment approaches

The family-centred, functional and DIR^®^/Floortime™ approaches were used most frequently. All six participants applied the family-centred approach to their telerehabilitation sessions. A majority of the activities and techniques that participants mentioned using were parent-driven and involved the family’s active participation in the preparation and facilitation of sessions.

Participant 1 reported that being involved in the sessions improved caregiver understanding of their child’s challenges:

‘All my therapy sessions were parent driven. … I was having a conversation with a parent to instruct them on how to do certain activities and focus on something specific.’ (P1, SLT, Western Cape)

Two participants used the functional approach to intervention (Owens, 2012), which can be considered appropriate for children with ASD. This approach helps clients apply the learnt skills to environments they are a part of in everyday life. One participant reported using the DIR^®^/Floortime™ approach (Greenspan & Wieder, 2008) with their clients and applying techniques such as mirroring and mediating finding solutions:

‘So the strategies that I used, so number one is Floortime approach that we use. So it is those types of techniques. So mirroring, you know, shapes, problem-solving. So it’s nontraditional speech therapy.’ (P3, SLT, KwaZulu-Natal)

#### Transitioning methods

As mentioned in the introduction, the COVID-19 pandemic resulted in the sudden transition from seeing clients face-to-face to conducting telerehabilitation. Four participants prepared their ASD clients for the transition by using social stories or discussing COVID-19 with the client. Two participants used show and tell as an interactive strategy for the client to welcome the participant into their home:

‘We did a social story for the kids themselves to explain what was happening with the lockdown, why therapy was no longer going to happen and why it would happen at home.’ (P2, SLT, Gauteng)

All participants mentioned the importance of finding a suitable environment for telerehabilitation to be effective. Two participants expressed the need to remove distractions whilst another two participants mentioned the importance of positioning to keep the client focused on the screen. This was required as most children with ASD cannot shift their attention or focus on a specific task with multiple distractions. This is because of their inability to understand which task is more critical (Mundy, Kim, McIntyre, Lerro, & Jarrold, 2016). Participants 2 and 6 determined appropriate spaces for therapy by conducting informal assessments of the house, either by requesting a video walkthrough of the house or by trial and error with each session to determine an appropriate spot:

‘The first thing that we asked for was a video walkthrough … reason being is we said we wanted to set up a space that a child specifically with autism then associates with therapy within the house.’ (P2, SLT, Gauteng)

Two participants reported the importance of keeping to a routine and using familiar songs. Therefore, these participants kept the same session times as face-to-face therapy, as this facilitated a smooth transition to telerehabilitation:

‘I tried to keep like the same time of the day that I normally see them.’ (P4, SLT, Gauteng)

‘I think it was just a combination of still being able to see their teachers, still hearing their familiar transition songs or music songs and kind of their parents getting them into a routine.’ (P5, SLT, KwaZulu-Natal)

#### Family collaboration

All six participants mentioned working closely with the family when conducting telerehabilitation. For participants with younger ASD clients (below the age of 4 years), the therapy sessions were more indirect and focused on parent coaching. The participants expressed positive experiences with parent coaching influencing the client’s generalisation and carrying over of the session’s aims:

‘They started using all therapy techniques every single day in those different areas, which allowed for stimulation to happen more often, and which in turn allowed for intervention to be more successful.’ (P2, SLT, Gauteng)

In addition, all participants reported that their caregivers were sent videos and home programmes to implement the skills targeted during the week. Caregivers played a more active role in telerehabilitation than in face-to-face sessions, as they were responsible for organising materials, facilitating the session and following up with the home programme between sessions:

‘Being at school, we’re working mainly with the teachers and the kids. But when you’re online, you need parents to be able to facilitate the session. So I actually really enjoyed the combination of working with the kids and their parents.’ (P6, SLT, Gauteng)

#### Maintaining attention

All participants incorporated the caregivers into their sessions to ensure that the clients maintained attention and concentration. Participant 5 mentioned that they collaborated with other professionals, such as occupational therapists, for advice about maintaining the client’s attention. Two participants mentioned using play-based activities with the clients as this kept them engaged:

‘So we use lots of game-based work play … always asked the mom and dad [to] leave a toy out that is their favourite. So, in the event that they lose attention, we grab that toy.’ (P6, SLT, Gauteng)

### Benefits of telerehabilitation

This section presents the subthemes that emerged related to the benefits of telerehabilitation. These include caregiver satisfaction and involvement, clinician satisfaction, the cost-effectiveness of telerehabilitation, accessibility to rural areas, clients’ progress in treatment and increased awareness of the role of the SLT, each of which is discussed in the following.

#### Caregiver satisfaction and involvement

All six participants reported a high level of caregiver satisfaction with telerehabilitation services. Participant 2 conducted a satisfaction survey with caregivers as her practice felt uncertain about whether telerehabilitation was suitable for their children. The participant found the caregivers with whom she was working were satisfied with services; however, they preferred face-to-face therapy as they required more reassurance when they were overwhelmed or stressed during telerehabilitation. On the other hand, two participants measured caregiver satisfaction by the gratitude expressed by the caregivers. Participant 4 reported caregivers felt grateful to have access to information and be involved in their child’s treatment during the pandemic. Furthermore, participants 4 and 5 mentioned caregivers being grateful for reducing the accessibility difficulties. Therefore, parents were less stressed with travel arrangements and attending different appointments across the city. The option of telerehabilitation was also viewed positively by parents as therapy could continue during the pandemic:

‘And then the kids that were unable to return continued online. … So I think that parents that continued online were really grateful that they had an option for educating their kids while they couldn’t come to school.’ (P5, SLT, KwaZulu-Natal)

Two participants mentioned that the success of telerehabilitation depends on the involvement of the family. Half the participants also noted that the caregiver’s involvement in telerehabilitation is greater than during face-to-face therapy:

‘It’s the same as you would have with the one-on-one therapy session, and I find that we end up with more parent involvement and understanding of therapy goals because they are there for each session.’ (P4, SLT, Gauteng)

During face-to-face therapy, there are separate roles between caregiver and SLT, and caregivers tend to observe instead of actively participating in the session. In telerehabilitation, the caregiver has to be engaged in the activities, prompt the child and apply the strategies learnt:

‘So it should be the same. But with face-to-face, families get away with a lot. Or sometimes they … [*are*] just like, “It’s not my job. It’s my job to be Mom; it’s your job to be therapists,” so they separated so much. Whereas when you are in their home via screen, they just … have to get involved.’ (P4, SLT, Gauteng)

Interestingly, there were mixed emotions regarding caregiver involvement. Participant 6 felt that occasionally caregivers would over-prompt the child, leading to difficulty determining whether the child is responding to the clinician’s prompt or the repetition of it:

‘I have a dad who would prompt the child every single time after I prompt the child. I think that also becomes challenging because they are getting over-prompted. Then you don’t really know if they are responding to your prompt or the repetition of the prompt.’ (P6, SLT, Gauteng)

This was supported by Participant 3 who shared that ‘helicopter parenting’ impacted on the sessions because of the caregiver taking over and ignoring the importance of providing a reinforcement to the child after the prompt.

‘If you do have parents who are helicopter parenting, they tend to take over, so that can be a disadvantage, but you know, obviously you need to give them a little briefing beforehand to say, “Fine, this is what I am planning.”’ (P3, SLT, KwaZulu-Natal)

In addition, Participant 2 felt that too many family members involved in the session causes the child to experience difficulty concentrating and engaging with the clinician. The findings suggest that caregiver satisfaction and involvement are high with telerehabilitation as the caregivers are responsible for the session, and the clinician serves as a facilitator.

‘Their own personal satisfaction of seeing that they had reached it with me just facilitating.’ (P3, SLT, KwaZulu-Natal)

However, there is a need for SLTs to set ground rules and provide training to avoid caregivers overstepping or applying ineffective communication strategies with their children.

#### Clinician satisfaction

The SLT’s views regarding telerehabilitation play a significant role in its success. All participants were satisfied with the opportunity to communicate with the caregivers and family. In addition, telerehabilitation provided the participants with the opportunity to observe the parent–child interaction in the home environment and provide meaningful real-time feedback to parents:

‘So I guess it opens up a nice opportunity to see how they live, what’s important to them, what they [*are*] keen on sharing with you. When you see them in your offices, it’s very different because they don’t always want to share certain things. When you [*are*] seeing them at home or online, you can see what’s important for their visual schedules.’ (P6, SLT, Gauteng)

Five participants agreed that they would continue providing telerehabilitation services as they were satisfied with the benefits. Some of these reasons include accessibility to outlying areas, more caregiver involvement and flexible schedules:

‘Look, telerehabilitation for me is the way of life; I’m one person and I have to travel between places that are 3 hours apart. So, for me, telerehabilitation brought another whole aspect to my practice. I can consult from two different places.’ (P6, SLT, Gauteng)

In contrast, Participant 5, who only used telerehabilitation during the lockdown period, did not agree that she would continue telerehabilitation despite identifying the benefits of using it:

‘I feel like it can be frustrating sometimes. When you get into it, and the kids are not responding, you’re not seeing the progress that you would face to face, then it can be quite disheartening. I think kids make it much more difficult.’ (P5, SLT, KwaZulu-Natal)

#### Cost-effectiveness of telerehabilitation

Prior to the lockdown, most participants provided SLT services at multiple sites such as schools, private practices and hospitals. This required the participants to incorporate travel costs and travel time into their monthly budget. Thus, many SLTs describe the cost-efficiency of telerehabilitation, as it was evident that reduced travel time and costs were an advantage.

‘I don’t have to use petrol to get to my office; I can do it from home. It’s very convenient. I could actually do it from anywhere if I do it on WhatsApp.’ (P1, SLT, Western Cape)

Affordable rates were reported to be another cost-effective aspect of telerehabilitation. Participants 2, 3 and 6 found that they could offer more affordable rates, thereby providing some financial ease for families. Telerehabilitation has shorter sessions, thus more affordable rates, lack of face-to-face activities and limited clinician-led treatment.

‘It’s cheaper compared to our normal rates. A lot more people are able to afford it, which allows us to increase our caseload. Our services are then given to people that need it and will generally not be able to afford it.’ (P2, SLT, Gauteng)

It was found that participants 2 and 4 were able to provide services to clients in outlying rural areas via telerehabilitation.

‘The same thing with access in the rural areas. … There aren’t a lot of therapists over there, where teletherapy has then allowed us to access those areas and provide services in those settings.’ (P2, SLT, Gauteng)

‘[*S*]olving the access problem in South Africa, as I think South Africa has a big access problem between the haves and the have-nots.’ (P4, SLT, Gauteng)

#### Clients’ progress in treatment

The findings demonstrate that telerehabilitation might be as effective as face-to-face interventions and a promising tool to consider utilising with children with ASD. Participant 4 reported progress seen in her clients; one client in particular has now become an effective communicator, and another has managed to continue mainstream schooling. This indicates that the shift to rehabilitation still resulted in significant progress:

‘I’ve had very good progress. And I think a lot of it has to do with the parents carrying through the home programs that we give because they are efficient, and they understand.’ (P4, SLT, Gauteng)

In addition, Participant 3 mentioned the clients with an already established relationship before telerehabilitation showed progress as much as they would have with face-to-face therapy:

‘I think it was more of the kids that I knew from their personality, because they didn’t need that one-on-one face time. That not only did they progress, but they progressed as much as versus seeing them.’ (P3, SLT, KwaZulu-Natal)

#### Increased awareness of the role of the speech-language therapist

Telerehabilitation provides the opportunity for SLTs to create awareness about their role in assessment and treatment. Participant 2 felt that caregivers often had a limited understanding of their child’s intervention goals and what needs to be prioritised in therapy, thus resulting in caregivers not identifying when their child with ASD experiences dysregulation or is attempting to communicate a need or a want. Participant 2 overcame this challenge by sitting down and discussing with caregivers the reasoning behind selecting short-term and long-term goals:

‘That’s why it is important to include the caregivers as well, because then you can sit down and explain the reasons as to why this needs to be focused on and why it is important.’ (P2, SLT, Gauteng)

Participant 4 reported that utilising telerehabilitation resulted in an increased awareness of the different healthcare professions. Participant 4 explained that by providing explanations for their child’s behaviour, there was an improved understanding regarding what is conducted in intervention:

‘It’s allowing parents to also get an education and just an awareness about therapies that are not known or understood by most of our population … the public isn’t overly aware, or there’s misconceptions about what a speech therapist is or what’s an occupational [therapist] is. So I think telehealth, then, just gives direct access for parents to enquire.’ (P4, SLT, Gauteng)

## Discussion

A variety of online assessment strategies are used by SLTs during telerehabilitation (Mashima & Doarn, 2008). In this study the participants used asynchronous methods of telerehabilitation for assessment more than synchronous methods, whilst still incorporating collaboration with family and other healthcare professionals. Informal assessments, including the use of videos, checklists and family reports were all favoured. The strategies follow those recommended by Oberleitner, Ball, Gillette, Naseef and Stamm (2006).

The above findings suggest that the family-centred approach applied in telerehabilitation played a significant role in developing treatment plans and goals that were functional for a child. This approach values the caregiver’s decision and places them in control with the SLT as the provider. This means that the caregiver’s needs are considered when planning treatment goals (Gibbs & Toth-Cohen, 2011). In addition to this, the functional approach (Owens, 2012) and DIR^®^/Floortime™ (Greenspan & Wieder, 2008) are important in the development and carryover of skills. All of these approaches highlight the need for family inclusion and participation in telerehabilitation sessions. This not only provides the caregiver with insight into their child’s difficulties but also supports carryover (Gibbs & Toth-Cohen, 2011). This is significant as poor awareness and knowledge regarding communication goals can lead to caregivers questioning the meaning of the treatment and may lead to them avoiding or adapting therapist-suggested activities (Gibbs & Toth-Cohen, 2011). Therefore, educating caregivers during sessions increased carryover in the child’s natural environment.

Coaching is another important part of family collaboration, particularly when working with younger clients and the only interaction between the SLT and client is indirect via the parent (Beiting & Nicolet, 2020). In this approach, the SLT is the planner and model, the caregivers are the implementers and the client is the participant. However, regardless of the approach, family involvement in speech-language therapy is important as caregivers can support speech-language intervention and improve their child’s quality of life by creating stimulating and rewarding environments outside the speech-language therapy clinic (Cohn et al., 2000). Family participation in therapy can also help keep children with ASD engaged during telerehabilitation, as they have knowledge of favourite toys, songs and games. The use of familiar objects and routines, as well as animated singing of songs, can help maintain attention and focus (Burgoyne & Cohn, 2020), all of which was confirmed by the participants in this study. A child with ASD is very attached to their familiar routines and will follow those routines; therefore, disruptions to the familiar routines can negatively affect their behaviour (Mirzaie, Jamshidian, & Hosseini, 2018). It was therefore important to try and set aside a consistent space within the home that would be associated with therapy and to hold onto familiar resources and routines when transitioning to telerehabilitation.

Caregiver satisfaction is a crucial factor to consider during telerehabilitation, as it is an indicator of the quality of service delivery (Kittredge, 2017) and influences the level of involvement a caregiver will have in their child’s treatment. Caregivers’ views of treatment and perceived success are important to inform the SLT’s decision-making on treatment methods and goals (Ong, 2019). Client satisfaction surveys opened a dialogue between caregivers and SLTs which show that the SLTs are listening and correcting issues, which contributes to the clinician–client relationship (Cleave, 2017). High levels of parent satisfaction with speech-language therapy conducted via telerehabilitation in the paediatric population during the COVID-19 pandemic were found by Tenforde et al. (2020). Grady (2002) found that telerehabilitation reduced general stress and saved time and money. The findings in this study are therefore consistent with the literature.

Oberleitner et al. (2006) found that healthcare professionals utilising telerehabilitation have a better understanding of what is happening to children in their natural environments at the time of their behavioural episode whilst simultaneously creating a need for family counselling. The benefits of seeing children in their own environment, albeit online, were confirmed by the participants of this study. Given the widespread use of teletechnology since the COVID-19 pandemic, it is likely that satisfaction with the usefulness of telerehabilitation will continue to improve with increased familiarity and use, as noted by the remaining five participants who continued using it in 2021. Therefore, there are high levels of clinician satisfaction regarding the use of telerehabilitation with ASD children.

Advantages such as scheduling efficiency, reduced travel time and reduced costs associated with both travel and visiting off-site clients are all cited in the literature (Beiting & Nicolet, 2020; Blaiser, Behl, Callow-Heusser, & White, 2013). It has been reported that telerehabilitation can increase access to therapy services for those who live in rural and remote areas (Beiting & Nicolet, 2020; Cason, 2009). In South Africa, there is a shortage of healthcare professionals as compared to the general population (Health Professions Council of South Africa, 2018), which makes telerehabilitation a good option. Unfortunately, therapy services in South Africa are unequally provided across its various socio-economic communities (Pillay, Tiwari, Kathard, & Chikte, 2020). The majority of SLTs practice in the Gauteng and Western Cape provinces of South Africa, despite these two provinces having the lowest percentage of persons with disabilities (5%) (Statistics South Africa, 2014). These services are still central. Therefore, the option of telerehabilitation may be an effective option to solve the challenge of individuals with disabilities in South Africa accessing healthcare.

## Conclusion and recommendations

In this sample in South Africa, telerehabilitation was successfully utilised to provide an alternative method to continue speech-language therapy services for children with ASD during the COVID-19 lockdown. Benefits to telerehabilitation were found to include caregiver satisfaction and involvement, clinician satisfaction, cost-effectiveness of telerehabilitation, accessibility to rural areas and increased awareness of the role of SLT. This study has many clinical implications. Firstly, the need to consistently provide information and support to caregivers to become facilitators during sessions is paramount. Encouraging and incorporating caregivers into the session promotes the generalisation of skills learnt and carryover into the child’s natural environments, thereby improving the quality of intervention provided to the clients. Information-sharing should include the reason for communication goals and the reasoning behind specific techniques. It is likely that telerehabilitation is here to stay. This warrants the need for SLTs to continue professional development in the area via reading, online courses and the use of social media platforms that serve as discussion groups to better prepare and support each other for alternative forms of service delivery. A further implication of this is the necessity to include telerehabilitation in undergraduate training programmes. Future research should explore the use of telerehabilitation for assessment of children with ASD, the use of telerehabilitation by therapists in the Departments of Education and Health and the use of telerehabilitation by SLTs working in rural contexts.

## Limitations

Participant recruitment was limited by not being able to gain permission from the Departments of Health and Education, which resulted in SLTs being recruited from the private sector and data being representative of only one context.
